# IRF7: role and regulation in immunity and autoimmunity

**DOI:** 10.3389/fimmu.2023.1236923

**Published:** 2023-08-10

**Authors:** Wei Ma, Gang Huang, Zhi Wang, Li Wang, Qiangguo Gao

**Affiliations:** ^1^ Department of Cell Biology, College of Basic Medical Sciences, Army Medical University (Third Military Medical University), Chongqing, China; ^2^ Department of Wound Infection and Drug, State Key Laboratory of Trauma, Burn and Combined Injury, Daping Hospital, Army Medical University (Third Military Medical University), Chongqing, China; ^3^ Department of Oncology, The Second Affiliated Hospital of Zunyi Medical University, Zunyi, Guizhou, China

**Keywords:** IRF7, IFN-I, IFN-III, COVID-19, immunity, autoimmunity

## Abstract

Interferon regulatory factor (IRF) 7 was originally identified as master transcriptional factor that produced IFN-I and regulated innate immune response, subsequent studies have revealed that IRF7 performs a multifaceted and versatile functions in multiple biological processes. In this review, we provide a comprehensive overview on the current knowledge of the role of IRF7 in immunity and autoimmunity. We focus on the latest regulatory mechanisms of IRF7 in IFN-I, including signaling pathways, transcription, translation, and post-translational levels, the dimerization and nuclear translocation, and the role of IRF7 in IFN-III and COVID-19. In addition to antiviral immunity, we also discuss the role and mechanism of IRF7 in autoimmunity, and the further research will expand our understanding of IRF7.

## 1 Introduction

Interferon regulatory factors (IRFs) are a family of master transcription factors, and although IRFs are recognized as transcriptional regulators of type I IFNs (IFN-I) and IFN-inducible genes, this family is now characterized as key factors in the regulation of many different processes, such as immunity, oncogenesis, metabolism, cell differentiation and apoptosis (1).

The IRF family consists of nine members, IRF1–9, previously reported in mammals. In addition, IRF10 has been identified in birds, dogs and teleost fish, and IRF11 is only found in teleost fish (2–4).

The IRF family has a conserved N-terminal region, and all members possess a helix-turn-helix DNA-binding domain (DBD), which contains five tryptophan repeats and recognizes the core DNA sequence of the 5’-GAAA-3’ tetranucleotide contained within the IFN-stimulated response elements (ISREs). The C-terminal regions of IRFs are diverse and related to distinct functions and contain two types of IRF-associated domains (IADs). The IAD mediates homo and heteromeric interactions with other IRF members, transcription factors, or cofactors to recognize DNA sequences and regulate gene transcription (5, 6).

IRF7 is a lymphoid-specific factor that is predominantly expressed in the cytoplasm of the spleen, thymus, and peripheral blood lymphocytes, such as B cells, plasmacytoid dendritic cells (pDCs) and monocytes. Although IRF7 was originally identified in Epstein-Barr virus (EBV) infection and characterized as a transcriptional regulator of IFN-I and IFN-stimulated gene (ISG), recent studies have revealed that IRF7 exerts a broad range of activities in different biological processes (7). In this review, as documented elsewhere, the structure of IRF7 is briefly summarized, however, we focus on the current knowledge about the regulation and function of IRF7.

## 2 The structure of IRF7

The IRF7 gene is located on chromosome 11p15.5 in humans and 7 F5 in mice, and four splicing variants of IRF7A, IRF7B, IRF7C, and IRF7H have been identified. IRF7A is the major splicing variant. Human IRF7A cDNA encodes a protein of 503 amino acid (aa) residues and mouse IRF7 consists of 457 aa residues. The nucleic acid homology of human and mouse IRF7 reaches 72.86% (8–10).

**Figure 1:**
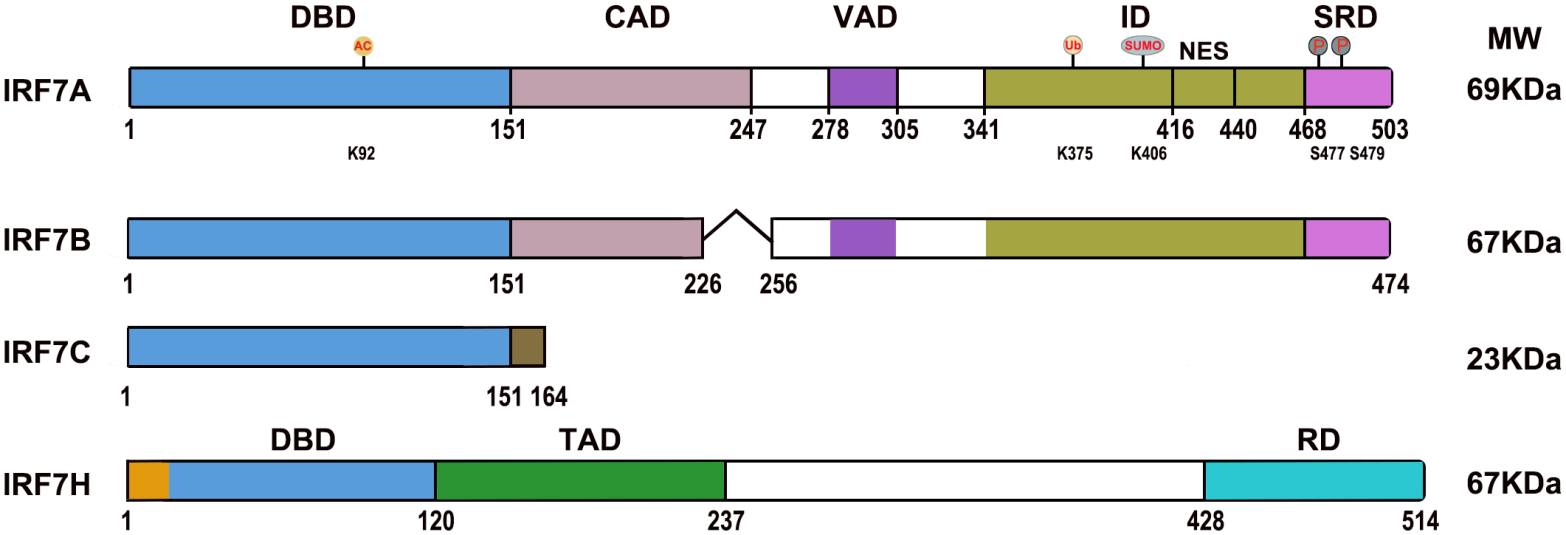
Diagrams of the IRF7 isoform domains. DBD, DNA binding domain; CAD, Constitutive activation domain; VAD, Virus activated domain; ID, Inhibitory domain; NES, Nuclear export signal; SRD, Signal response domain; TAD, Transactivation domain; RD, Regulatory domain. “˄” represents deleted regions. AC, Acetylation; Ub, Ubiquitination; SUMO, SUMOylation; P, Phosphorylation; K92, lysine 92; K375, lysine 375; K406, lysine 406; S477, Serine 477; S479, Serine 479.

IRF7 (IRF7A) has a distinctive multiple domain structure in its C-terminal region, except for the DBD in the N-terminal region, present in all IRF members. With the deletion mutations of IRF7, a constitutive activation domain (CAD) is located between 151–246 aa adjacent to the conserved DBD (1-150 aa), which maintains the activity of IRF7, and a nuclear localization sequence may be present in the region between 1 and 246 aa. The region located between 278 and 305 aa contributed to the virus activated domain (VAD), which is indispensable for the activation of IRF7, and the sequence collaborates with the C-terminal signal response domain for maximal response to virus infection, and nuclear translocation may be controlled by the region. The inhibitory domain (ID) located between 341 and 467 aa interferes with the transactivation function of IRF7, but primarily within the region of 416–467 aa, and this sequence contains an efficient nuclear export signal (NES), deletion of the region of 417 and 440 aa, IRF7 failed to nuclear translocation. The second inhibitory domain is located between 341-415 aa, which may affect the DNA binding and/or transactivation activity of IRF7. The signal response domain (SRD) or accessory inducibility region located at the C-terminal end between 468 and 503 aa mediates IRF7 dimerization and contains a serine-rich domain, and the phosphorylation of Ser 477 and Ser 479 is vital for IRF7 because the substitution of S477 and S479 completely abrogated cytoplasmic to nuclear translocation. Nuclear export of IRF7 requires sequences within VAD, ID and phosphorylation of S477 and S479 (11, 12) (**Figure 1**). IRF7 is rich in the PEST (proline (P), glutamic acid (E), serine (S), and threonine (T)) sequence due to its very short half-life, and its stability is controlled by the ubiquitin–proteasome system (9, 13).

IRF7B lacks 29 aa (from G227 to T255) in the middle of the CAD difference from IRF7A. IRF7C consists of 164 amino acid residues and unique 13 aa sequence at the C-terminus difference from IRF 7A; it is not only a dominant negative regulator that blocks the activation of IFN for IRF7A and IRF7B but is also associated with EBV transformation of human primary B cells as well as EBV type III latency and plays a role in the development of lymphoma (10, 12, 14). The spliced variant IRF7H encodes 514 aa and differs from IRF7A by 18 aa at the N-terminal region, displays functional similarity to IRF3, plays an important role in regulating the expression of the *IFNA* gene (8) (**Figure 1**).

## 3 The function of IRF7

IRF7 has a wide range of functions. Herein, we present a review regarding its function and regulation in immunity. We also discuss the latest relevant research in autoimmunity and oncogenesis toward understanding IRF7 beyond its role in immunity.

### 3.1 IRF7 in immunity

IRF7 is involved in the regulation of mouse and human IFN-I/III, especially IFN-I.

#### 3.1.1 IRF7 and IFN-I

##### 3.1.1.1 IRF7-mediated IFN-I signaling pathway

IFN-I, mainly IFN-α and IFN-β, is a critical pleiotropic cytokine for immunity against the viral response and has been characterized as triggering antiviral states in cells and potentiating adaptive immune responses. IRF7 is the master regulator of IFN-I immune responses, not only regulating further expression of IFN-β but also triggering IFN-α production (15). The specific knockout of IRF7 in mouse pDCs almost loses the ability to produce IFN-α (16, 17), and the deficiency of IRF7 in humans also significantly inhibits the production of IFN-α (18).

**Figure 2:**
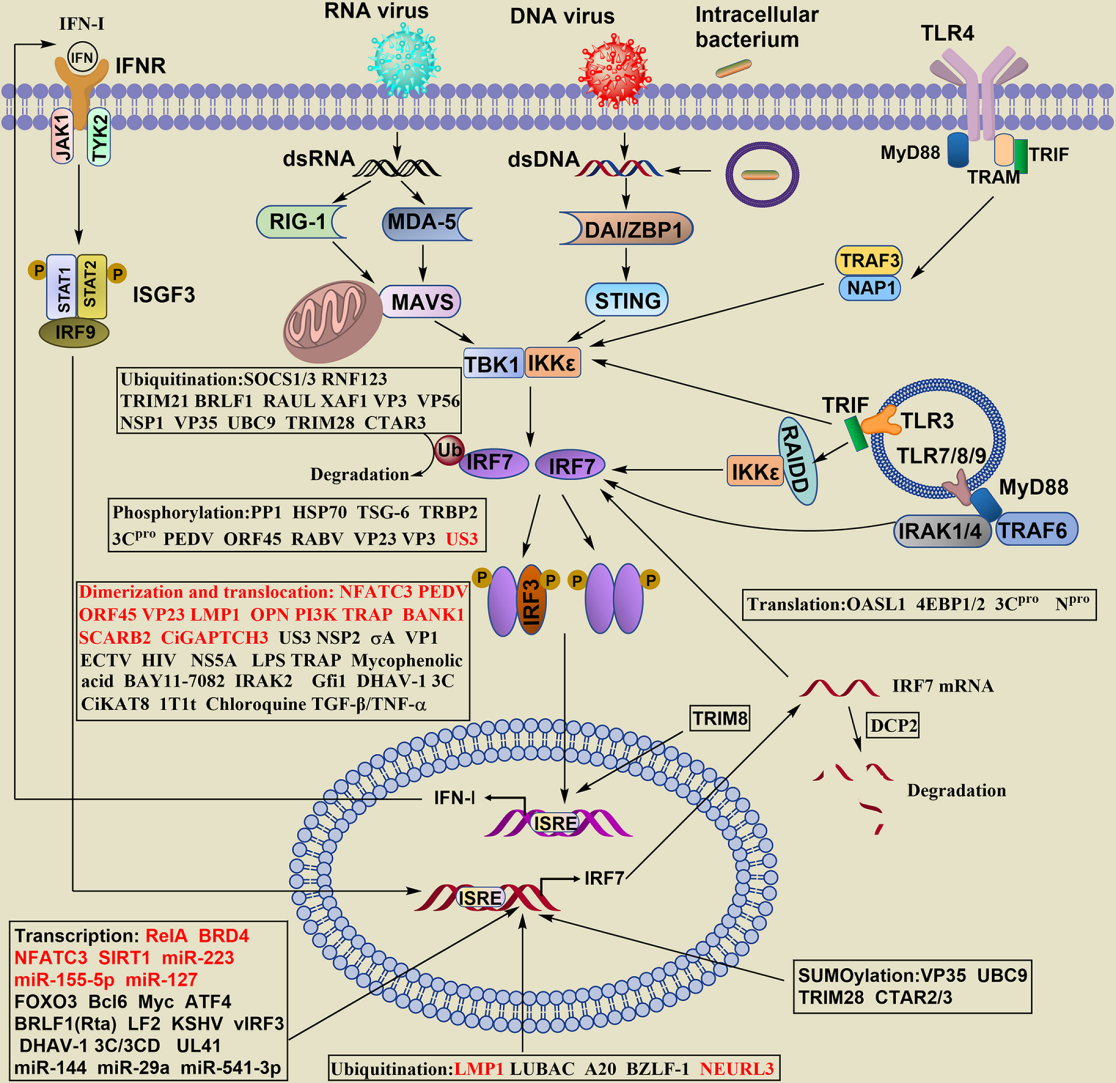
Regulation of IRF7 signaling pathway in the production of IFN- I. The stability of IRF7 can be regulated at the levels of transcription, translation, posttranslation, epigenetics, the dimerization and nuclear translocation. The regulatory levels are highlighted in boxes, words in red of the boxes indicate positive regulators, and words in black show negative regulators.

The production of IFN-I has been widely reported. Briefly, after viral infection and others, IRF7 in the cytosol is activated by distinct types of innate pattern recognition receptors (PRRs), and the PRRs associated with IRF7 can be classified as cytosolic and transmembrane signaling. The phosphorylation of IRF7 and IRF3 occurs mainly by innate immune cells contacting virus-specific antigenic substances (DNA, RNA) through PRRs and then by intracellular signaling molecules (MAVS, STING, TBK, IKKϵ, etc.). Dimerized IRF7 or IRF3/IRF7 can translocate into the nucleus to initiate expression of the IFN-I gene (**Figure 2**). Honda and Taniguchi reported that the homodimer of IRF7 or the heterodimer of IRF7/IRF3, rather than the homodimer of IRF3, is more important for the production of IFN-I under viral infection (19).

For cytoplasmic signals, RIG-I or MDA5 recognize viral RNA, DNA sensors also known as DAI (DNA-dependent activator of IRFs) or ZBP1 (Z-DNA-binding protein 1), including DDX (dead-box polypeptide) 41, MRE11 (meiotic recombinant 11 homolog A), IFI (IFN-γ induction) 16 and cGAS (loop CMP-AMP synthase), to detect cytosolic DNA. The induction of IFN-α in cytosolic DNA is required for both IRF3 and IRF7.

Transmembrane Toll-like receptor (TLR) signaling can be divided into MyD88-dependent and TRIF-dependent (MyD88-independent) pathways. TLR7/8/9 is expressed in the membrane of endosomes and phagosomes and employs a MyD88-dependent pathway to induce IFN-I. IRF7 is activated and translocated to the nucleus via the signaling cascade of MyD88-IRAK4-IRAK1-TRAF6, leading to the production of large amounts of IFN-I in pDCs.

TLR3 is mainly localized to endosomes and uses the adaptor protein TRIF to recruit TANK-binding kinase (TBK)1 to endosomes and phagosomes, activating IRF3 and IRF7 to induce the expression of IFN-I. Additionally, the adaptor molecule adaptor molecule RIP associated ICH-1/CED-3-homologous protein with a death domain (RAIDD) interacts with IKKϵ and IRF7 but not TBK1, mediates IRF7 phosphorylation and triggers IFN-I production (20).

TLR4 is located on the cell surface and is the only member in the TLR family that transduces signals by two pathways. The MyD88-dependent pathway requires IRF3 rather than IRF7. Endocytosed TLR4 activates the adaptor protein TRAM-TRIF, recruits TRAF3, NAP1 and TBK1/IKKϵ, and activates IRF7 to induce IFN-β and IFN-α4 but not other IFN-α genes.

IRF7 is required for IFN priming at an early stage, which restricts not only the acute infection of herpesvirus families but also chronic gamma herpesvirus infection. IRF7 suppresses the establishment of latency and viral reactivation in the peritoneal cavity and attenuates viral reactivation in the spleen, promoting the expression of select MHV68-restircting ISGs in peritoneal cells (21).

IRF7 is also required for IFN-I amplification at later stages, and the secondary, larger wave of IFN-I expression depends on the positive regulatory loop between IRF7 and IFN-I. This pathway regulates the transcription of IRF7 (**Figure 2**).

##### 3.1.1.2 The regulation of IRF7 in IFN-I

For the key role of IRF7 in IFN-I production, the balance between its activation and repression needs to be delicately maintained. The regulation of IRF7 is complex and involves many positive and negative feedback mechanisms. The stability of IRF7 can be regulated by various mechanisms at the transcriptional, translational, posttranslational, epigenetic levels, such as phosphorylation, ubiquitination, SUMOylation, acetylation, et al., and the dimerization and nuclear translocation of IRF7 (**Figure 2**).

###### 3.1.1.2.1 Transcriptional level

The transcription of IRF7 has two distinct pathways: IFN-triggered and IFN-independent signaling pathways. IFN signals through its receptor to induce the phosphorylation of STAT1 and STAT2, which results in the formation of ISGF3 and then promotes the transcription of IRF7 by binding directly to the IRF7 interferon (IFN)-stimulated response element (ISRE) and IRF-binding element (IRFE), which is IFN-triggered signaling (22, 23). Virus-induced formation of a virus-activated factor complex (formed by IRF7, IRF3 and p300/CREB-binding protein) directly binds to the IRF7 ISRE and IRFE and stimulates the intrinsic transcriptional activity of IRF7, and this induction is independent of the IFN-triggered pathway (24, 25). Some factors can regulate IRF7 transcription positively or negatively.

Phosphorylated RelA and bromodomain containing (BRD)4 induced by respiratory syncytial virus (RSV) can bind to the IRF7 promoter, triggering the IRF7-RIG-1 amplification loop for IFN-I/III expression (26, 27). Transcription factor nuclear factor of activated T cells (NFATC) 3 selectively binds to the autoinhibitory domain (373-443 aa) of IRF7, promotes transcription and nuclear translocation, and enhances CpG DNA–induced IFN-α production in pDCs (28).

FOXO3 is a negative regulator of IRF7 transcription, and the ternary complex consisting of FOXO3, nuclear corepressor 2 (NCOR2) and histone deacetylase 3 (HDAC3) on the Irf7 promoter enhances the closed chromatin structure and represses Irf7 expression and *Ifnb1* production (29). B-cell lymphoma (Bcl)6, interacting with NCOR2 and HDAC3, binds to IRF7 loci and restrains its transcription (30). Myc forms a repressor complex together with NOCR2 and HDAC3 to reduce the expression of IRF7 through histone deacetylation (31). Activating transcription factor (ATF) 4 inhibits the transcription of IRF7 by regulating its promoter. However, IRF7 increases the expression and function of ATF4 directly, and cross-regulation between IFN and integrated stress responses is mediated by the reverse correlation between ATF4 and IRF7 (16).

Viral derived proteins also inhibit the transcription of IRF7, such as Epstein-Barr virus immediate early protein BRLF1 (Rta) and LF2, Kaposi´s sarcoma-associated herpesvirus (KSHV) viral homologs of IRF3 (vIRF3), duck hepatitis A virus type 1 (DHAV-1) 3C and 3CD protein, and duck enteritis virus tegument protein UL41 (17, 32–35).

Elevated expression of the decapping enzyme Dcp2 induced by viral infection and double-stranded RNA treatment inhibited IRF7 mRNA stability and protein accumulation (36).

MicroRNAs regulate IRF7 expression by modulating mRNA stability. miR-223 and miR-155-5p enhance IRF7 expression by targeting FOXO3 (37, 38), miR-127 promotes IRF7 expression by inhibiting the signal-dependent turnover of the Bcl6 coregulator (30), miR-144 negatively regulates IRF7 activity by suppressing the TRAF6-IRF7 signaling axis (39), and miR29a inhibits the expression of IRF7 and TRAF6 (40). miR-541-3p promotes the replication of porcine reproductive and respiratory syndrome virus (PRRSV) 2 by negatively regulating the transcription of IFN-I by targeting IRF7 (41).

###### 3.1.1.2.2 Methylation

As a major epigenetic modification, CpG hypermethylation in the promoter region can lead to the inactivation of specific gene expression and function. Zhang Y et al. checked the 5-methylcytosine level of the CpG island infected by infectious bursal disease virus (IBDV) and found that CpG island methylation in the IRF7 promoter regions was substantially decreased (42). Rezaei R analyzed the methylation status of CpG sites of the IRF7 promoter in peripheral blood mononuclear cells (PBMCs) of systemic sclerosis (SSc) patients and found that hypomethylated CpG2 was associated with increased disease risk (43). The zinc finger CXXC family epigenetic regulator CXXC5 maintains the hypomethylation states of the CpG-containing island (CGI) within the Irf7 gene by recruiting the DNA demethylase Tet2 (44). Cigarette smoke condensate decreases the expression of IRF7 mRNA with increased CpG methylation of its promoter region in PBMCs (45).

###### 3.1.1.2.3 Translational level

Although the regulation of transcription levels can control gene expression from the source, the slower nuclear pathways for mRNA synthesis and transport affect the effect. Translation control of gene expression enables cells to respond quickly to external and internal triggers or clues, and translational control of IRF7 is vital to the induction of IFN-I production.

By binding to the 5’ untranslated region (UTR) of IRF7, 2ʹ-5ʹ-oligoadenylate synthase-like protein 1 (OASL1) prevents the loaded 43S preinitiation complex from passing the UTR and inhibits the translation of *Irf7* mRNA. The translational repressors 4E-BP1 and 4E-BP2 also suppress the translation of *Irf*7 mRNA by recognizing the 5’UTR, negatively regulating the production of IFN-I (46, 47).

The 3C protein of enterovirus 71 mediates the cleavage of IRF7 at the Q189-S190 site in the CAD domain and inhibits IRF7 function (48).

N^pro^ of classical swine fever virus (CSFV) interacts with IRF7 through its Zn-binding domain and negatively regulates IRF7 protein expression but not mRNA expression in pDCs (49).

###### 3.1.1.2.4 Posttranslational modification of IRF7

Posttranslational modifications (PTMs) are important epigenetic mechanisms regulating various biological processes, with the key advantages being that PTMs regulate faster and require less energy than protein regulation. These modifications include phosphorylation, acetylation, ubiquitylation, SUMOylation, and neddylation.

####### 3.1.1.2.4.1 Phosphorylation

After pathogenic infection, IRF7 changes from an inactive to activated state by phosphorylation, and the activation of IRF7 requires two phosphorylation events. IRF7 needs at least one phosphorylation event at S477, S479, S483 and S487 in addition to phosphorylation of both S471 and S472 (50).

The phosphorylation of IRF7 is linked to PRR pathways. TLR7/8 antagonists reduce Helicobacter pylori infection-mediated IRF7 phosphorylation (51). Kinases such as IKKϵ, TBK1, IRAK1 and IKKα are responsible for the phosphorylation of IRF7 in a cell type-specific manner (52, 53).

In addition to the direct signaling kinases that regulate the phosphorylation of IRF7, other molecules may act by regulating these signaling molecules.

The phosphatase protein phosphatase (PPP)1 can target four key phosphorylation sites (Ser471, -472, -477 and -479) of IRF7, attenuate the phosphorylation level and DNA-binding activity stimulated by IKKϵ, and inhibit IRF7-mediated IFN-I immune responses (54). HSP70 interferes with IKKϵ-mediated IRF7 phosphorylation via simple competition for IRF7 binding (55), and the anti-inflammatory factor TNF-a–stimulated gene 6 (TSG-6) downregulates IRF7 activity by suppressing its phosphorylation mediated by CD44 (56). TARBP2 promotes the K48-linked polyubiquitination and proteasome-dependent degradation of TRAF6 and weakens the interaction between TRAF6 and IRF7, thus inhibiting the phosphorylation of IRF7 (57).

Viral proteins can interact with IRF7 and suppress its phosphorylation, dimerization and nuclear translocation. For example, Seneca Valley Virus (SVV) protein 3C^pro^ (58), the 1-55 region of porcine epidemic diarrhea virus (PEDV) membrane protein (59), Kaposi´s sarcoma-associated herpesvirus (KSHV) immediate-early proteins ORF45 (60), the negative single stranded RNA rabies virus (RABV) phosphoprotein P (61–63), and Marek´s disease virus VP23 protein (64), enterovirus D68 VP3 (65) et al. Unique-short kinase (Us)3 of Marek´s disease virus or duck enteritis virus can induce IRF7 phosphorylation by interacting with its activation domain but suppress dimerization and nuclear translocation (66, 67).

####### 3.1.1.2.4.2 Acetylation

The acetylation of transcription factors can affect DNA binding affinity, stability, degradation and protein-protein interactions (68). IRF7 is subject to acetylation by the histone acetyltransferase (HAT) of the PCAF (p300/CBP Associated Factor)/GCN5 family. The unique lysine residue target for acetylation is located in the DNA binding domain at lysine 92 (K92), and acetylation inhibits IRF7 DNA binding and decreases transcriptional activity (69) (**Figure 1**).

The acetyltransferase KAT8 in grass carp (CiKAT8) directly interacts with IRF3/7 via the acetyltransferase domain (CiKAT8-∆264-487), blocks the interaction between IRF3/7 and ISRE, and inhibits IFN-1 expression (70).

Liquid–liquid phase separation (LLPS) is a key mechanism for transcriptional regulation. NAD-dependent protein deacetylase sirtuin-1 (SIRT1) directly deacetylates K39 and K77 on IRF3, and K45 and K92 on IRF7 are located in the DBD, promoting LLPS of IRF3/7 and enhancing IFN-1 transcription (71).

####### 3.1.1.2.4.3 Ubiquitination

Ubiquitination can either activate or inactivate/degrade IRF7. Nondegradative ubiquitination is involved in the activation of IRF7 and is a prerequisite for its phosphorylation. Epstein-Barr virus latent membrane protein (LMP) 1 promotes the phosphorylation and nuclear translocation of IRF7 through ubiquitination (72, 73). IRF7 binds to the ISRE of the LMP1 promoter and forms a positive regulatory loop between IRF7 and LMP1 (74). The linear ubiquitin assembly complex (LUBAC) of the LMP1 downstream target, antiapoptotic factor deubiquitinase A20 induced by LMP1, and EB virus BZLF-1 negatively regulate the transcriptional activity of IRF7 via LMP1-stimulated IRF7 ubiquitination (75–77). The E3 ubiquitin ligase NEURL3 ubiquitinates the K63-linked polyubiquitin chain on IRF7 lysine 375 (K375) (**Figure 1**), impairs the association of IRF7 with histone deacetylase (HDAC)1, and enhances the transcription of IFN-I and ISGs (78).

In addition to activating IRF7, ubiquitin-mediated degradation of IRF7 is an effective mechanism to regulate its activity. Suppressor of cytokine signaling (SOCS) 1/3 mediates the degradation of IRF7 by lysine 48-linked polyubiquitination through the SH2 domain and suppresses IFN-I production (79). Ring finger protein (RNF)123 promotes TLR-3- and IRF7-mediated IFN-I expression by ubiquitination and proteasomal degradation of SOCS1 (80). TRIM21 (Ro52) cooperates with the Fas-associated death domain (FADD) to enhance TRIM21 ubiquitin ligase activity, promote the ubiquitination and degradation of IRF7, and repress its phosphorylation and transcriptional activities. FADD and TRIM21 constitute a negative feedback loop of the IFN-α pathway (81). RAUL (RTA-associated ubiquitin ligase) directly catalyzes lysine 48-linked polyubiquitination of IRF7, promotes ubiquitin-proteasome dependent proteolysis (82). and the XAF1-XIAP-KLHL22 axis inhibits IFN-I induction through CUL3-KLHL22, which directly ubiquitinates IRF7 (83).

In addition to inhibiting the phosphorylation and nuclear translocation of IRF7, enterovirus D68 VP3 disturbs the TRAF6-triggered K63-ubiquitination of IRF7 and blocks its activation (65). KSHV BRLF1 (Rta) promotes polyubiquitination and proteosome-mediated degradation of IRF7 (84), Rotavirus nonstructural protein NSP1, acting as a putative E3 ubiquitin ligase, induces proteasome-mediated degradation of IRF7 and suppresses the expression of IFN-β (85).Grass carp reovirus (GCRV) VP56 promotes the K48-linked ubiquitination of phosphorylated IRF7, inhibits IFN expression (86).

####### 3.1.1.2.4.4 SUMOylation

Small ubiquitin-like modifier (SUMO) is a ubiquitin-like small protein that can be conjugated onto target proteins to increase the functional repertoire of the eukaryotic proteome (87). TLR and RIG-I/MDA-5 signaling mediate the SUMOylation of IRF7 but not the IFN-activated JAK/STAT pathway in response to vesicular stomatitis virus (VSV) or encephalomyocarditis virus (EMCV) infection. SUMOylation is independent of phosphorylation, and lysine 406 (K406) of IRF7 is the SUMO conjugation site (88) (**Figure 1**). The encoded protein VP35 by filoviruses, such as Ebola virus and Marburg virus, not only interacts with IKK-ϵ and TBK-1 to block the activation of IRF7 but also promotes PIAS1-mediated SUMOylation of IRF7 to inhibit the transcription of the IFN gene (89, 90). The LMP1-mediated SUMO conjugating enzyme UBC9 not only promotes SUMOylation but also negatively regulates the chromatin binding of IRF7. TRIM28, the IRF7-specific SUMO E3 ligase, increased the SUMOylation of IRF7 during viral infections, inhibiting its transcriptional activity (91). Bentz G et al. showed that LMP1 C-terminal activating region (CTAR)3, in cooperation with CTAR2, induces the SUMOylation of IRF7 and decreases its turnover rate and transcriptional activity (88, 92).

####### 3.1.1.2.4.5 Neddylation

Neddylation is a ubiquitin-like posttranslational protein modification that is indispensable for the production of RNA virus-induced IFN-α. IRF7 was identified as the neddylation substrate, and neddylation was first detected in zebrafish (93). Neddylation cannot promote RNA virus-induced IRF7 expression but enhances the transcriptional activity and nuclear translocation of IRF7 (94).

###### 3.1.1.2.5 Dimerization and nuclear translocation of IRF7

After phosphorylation, dimerized IRF7 in the nucleus can bind to the promoter regions of target genes to activate transcription with the help of other coactivators, and the regulation of dimerization and translocation of IRF7 is a critical step in the production of IFN-I.

In addition to NFATC3, PEDV membrane protein, ORF45, VP23 and LMP1 promote the dimerization and nuclear translocation of IRF7, and other viral proteins, such as Us3, nonstructural protein(NSP) 2 of H1N1 influenza A virus, avian reovirus σA protein and the structural protein VP1 of chicken anemia virus, decrease the dimerization and nuclear translocation of IRF7 (95–97).

Viral infection affects the nuclear transport of IRF7. Infection with Ectromelia virus (ECTV) decreases the nuclear translocation of NF-κB, IRF3 and IRF7 in murine GM-CSF-derived bone marrow cells (98). HIV suppresses AKT phosphorylation to inhibit the translocation of IRF7 into the pDC nucleus (99). In HCV-positive hepatoma cells, stimulation with the TLR7 ligand increases IRF7 nuclear translocation (100). In contrast, in hepatocytes, HCV infection disturbs the phosphorylation of STAT1, blocks the nuclear translocation of IRF7 through the NS5A protein and inhibits the expression of IFN-α (101, 102). LPS, the major component of the outer membrane of gram-negative bacteria, suppresses virus-mediated phosphorylation and nuclear translocation of IRF3/IRF7 (103).

The components of the signal transduction pathway can regulate the nuclear translocation of IRF7. Intracellular osteopontin (OPN) and PI3K selectively promote IRF7 nuclear translocation and subsequent type I IFN production (104, 105). Tartrate-resistant acid phosphatase (TRAP) decreases the phosphorylation of OPN and then blocks the nuclear translocation of IRF7 and p65 (106). Mycophenolic acid, the metabolic product of mycophenolate mofetil, inhibits IRF7 nuclear translocation and IFN-α production by suppressing PI3K-AKT activity (107). An inhibitor of the IKK complex, BAY11, (E)-3-(4-methylphenylsulfonyl)-2-propenenitrile (BAY11-7082), has the ability to repress the nuclear translocation of IRF7 and IFN-α production (108). IL-1R-associated kinase (IRAK) 2 decreases the nuclear translocation of IRF7 in response to stimulation with TLR9 ligands (109). The transcriptional repressor growth factor independence 1 (Gfi1) prevents spontaneous SLE by negatively regulating TLR7 signaling and nuclear translocation of IRF7 in DCs (110). B-cell scaffold with ankyrin repeats (BANK)1 can increase the expression and nuclear translocation of IRF7 upon TLR7 stimulation in B cells and promote IgG production in autoimmune disease (111). Scavenger receptor class B member 2 (SCARB2) deficiency in pDCs impairs nuclear translocation of IRF7 and decreases endosomal translocation of TLR9 (112). Some small molecules affect the nuclear translocation of IRF7 in pDCs. 1T1t, the small molecule CXCR4 ligand, inhibits TLR-7 mediated IFN-α production through blocking the phosphorylation and nuclear translocation of IRF7 (113). Chloroquine, an endosomal inhibitor, blocks TLR signaling, decreases the expression and nuclear translocation of IRF7 and production of IFN-α (114, 115). TGF-β and TNF- α synergistically impaired IFN- α production of TLR-activated pDC through blocking the expression and nuclear translocation of IRF7 (116).

Additionally, cord blood pDCs do not function as their adult counterparts, especially in terms of the defect in IFN production, due to the deficiency in IRF7 nuclear translocation (117). Toxoplasma gondii inhibits virus-induced nuclear translocation of IRF7 via the tyrosine kinase ROP16 in pDCs (118). Ctenopharyngodon Idella (Ci) GAPTCH3 directly interacts with CiSTING and enhances the phosphorylation and nuclear translocation of CiIRF7 (119), and the DHAV-1 3C protein inhibits the nuclear translocation of IRF7 (35).

In the nucleus, TRIM8 stabilizes phosphorylated IRF7 and protects it from peptidyl-prolyl isomerase Pin1-induced degradation in primary pDCs (120).

#### 3.1.2 IRF7 and IFN-IIIs

IFN-IIIs are also called lambda IFNs (IFNλs), produced by cells of hematopoietic origin or epithelia at barrier surfaces, and include four members in humans (IFNλ1 or IL-29, IFNλ2 or IL-28A, IFNλ3 or IL-28B, IFNλ4) and two in mice (IFNλ2 or IL-28A, IFNλ3 or IL-28B). IFN-III genes are located on chromosome 19 in humans and chromosome 7 in mice, though IFN-IIIs share homology, expression patterns, antiviral functions and signaling cascades with IFN-Is, some features distinguish the two IFNs: (i) the initiation time, IFN-IIIs are the earliest and predominant IFNs during virus infection, mediate front-line antiviral defense without activating inflammation, while IFN-Is come up later to enhance viral resistance and proinflammatory responses (121, 122). (ii) the distinct heterodimeric receptors, the IFNAR receptor complex (IFNAR1 and IFNAR2) is ubiquitously expressed, and is bound to IFN-Is, while the IFNLR receptor complex (IFNLR1 and IL10Rβ) is confined expressed on epithelial cells and a subset of myeloid lineage leukocytes, and is bound to IFN-IIIs (122). (iii) the signaling pathway, in addition to activating the same JAK1 as IFN-Is, IFN-IIIs also activate JAK2, and MAPKs are required for the antiviral activity of IFN-IIIs but not IFN-Is (123); contrary to mitochondrial MAVS for activate IFN-Is, peroxisome MAVS induces IFN-IIIs expression only.(iv) the signaling kinetics of ISG induction, IFN-Is mediate rapid and short-lived expression of ISGs with higher magnitude, while IFN-IIIs induce persistent expression of ISGs with lower magnitude (124).

In addition to IFN-I, IRF7 is also involved in IFN-III, IFNλ1 gene is regulated by virus-activated IRF3 and IRF7 like IFN-β gene is, while IFNλ2/3 gene is controlled by IRF7 like IFN-α gene is (125), and IRF7-mediated IFN-III plays a major role in antiviral protection of epithelial barriers due to restricted expression of the receptors (126, 127).

IRF7 mediates the induction of IFN-III caused by viral infection. Senecavirus A (SVA) is recognized by RIG-1/MDA-5 receptors in porcine kidney PK-15 cells and then activates downstream IRF7- but not IRF3-mediated signaling pathways, causing the upregulation of IFN-λ1, IFN-λ3 and related ISGs (126). 5-aza-2-deoxycytidine (5-AZA-CdR), a DNA-demethylating agent, induces IFN-III but not IFN-I production by the MDA5/MAVS/IRF7-dependent or JAK-dependent “viral mimicry” pathway (128). Rotavirus UK-like UKtc NSP1 reduces IRF7-dependent transcription at high IRF7 concentrations and inhibits IFN-III induction in intestinal epithelial cells (129).

With a synergistic interaction between IRF7 and p65 at the TA-repeat polymorphism (rs72258881) of the IFN-λ3 promoter, IRF7 is sensitive to changes in DNA phasing and mediates the transcription of IFN-λ3 (130).

Clinically, the upstream gene of IRF7 is reduced in patients with atopic dermatitis with a history of eczema herpeticum (ADEH^+^) compared with healthy subjects and patients with ADEH- after HSV-1 exposure, thus affecting the expression of IFN-IIIs (131). Med23, a subunit of the Mediator multiprotein complex, is specific to HSV-1 and regulates the induction of IFN-λ by interacting with and enhancing the activity of IRF7 (132).

#### 3.1.3 IRF7 and COVID-19

The role of IRF7 in COVID-19 is conflicting. On the one hand, IRF7 is protective against viral infection. Campbell TM et al. reported that IRF7-deficient patients are prone to severe respiratory viral infections, with influenza and COVID-19, due to impaired type I and III IFN expression in both pDCs and respiratory epithelial cells (133–135). Patients with severe COVID-19 at early time points show decreased levels of type I and III IFN (136). TLR3- and TLR7-dependent production of IFN-I by pDCs and respiratory epithelial cells is essential for host defense against SARS-CoV-2 (137). Zhang et al. sequenced the genome or exome of 659 patients with COVID-19 to test 118 rare nonsynonymous variants of 13 human loci that underlie life-threatening influenza pneumonia. Autosomal-recessive and autosomal-dominant deficiencies of IRF7 are involved in the TLR3- and IRF7-dependent induction and amplification of IFN-I (138), and TLR7 together with IRF7 combat COVID-19 by the large amounts of IFN-I produced by pDCs (139). However, Povysil G et al. declared that they did not observe the enrichment of predicted loss-of-function (pLOF) variants in severe cases relative to population controls or mild COVID-19 cases postulated by Zhang et al. (140). The high expression of SCV2-miR-ORF1ab-2-5p, one of the four unique microRNA-like small RNAs encoded by SARS-CoV-2, inhibits the host IFN response by targeting IRF7 and IRF9 (141).

However, IRF7 may exacerbate the progression of COVID-19. IFN-I, IRF7 and ISGs are highly expressed in the oropharyngeal cells of SARS-CoV-2-positive patients (142), and strong IFN- I responses in patients with severe COVID-19 (143), for IFN- I may aggravate TNF- and IL-1-driven inflammation. With the analysis of the most frequent comorbidities in COVID-19, the authors found that the hub protein IRF7 is upregulated in COVID-19 patients, which is associated with the pathogenesis of diabetes mellitus and lung cancer (144). IRF7 is strongly hypomethylated in SARS-CoV-2 individuals, and IRF7 DNA methylation signatures may differentiate patients with SARS-CoV-2 infection from uninfected individuals and predict COVID-19 disease severity (145). The role of IFN-1 in COVID-19 may depend on mild versus severe and early versus late disease.

### 3.2 IRF7 and autoimmune diseases

Autoimmune diseases refer to diseases caused by the body’s immune response to its own antigen and damage to its particular tissue or system, which can be classified as systemic autoimmune disease (SAD) and organ-specific autoimmune disease. Upregulation of IFN-I is a hallmark of SAD, and the continuous increase in IFN-I/III may be accompanied by clinical manifestations and disease activity (146, 147). As the master regulator of IFN-I/III, IRF7 has a dual role as a protector and cause of autoimmune diseases. In published articles, decreased expression was observed in multiple sclerosis (MS)/experimental autoimmune encephalomyelitis (EAE) and rheumatoid arthritis (RA), while increased expression of IRF7 was observed in patients with systemic lupus erythematosus (SLE), systemic sclerosis (SSc), autoimmune pancreatitis (AIP), autoimmune thyroid diseases (AITD) and diabetes compared to healthy controls (**Table 1**).

**Table 1:** IRF7-related autoimmune diseases.

Diseases	Mechanisms and Functions	References
Multiple sclerosis/EAE	IRF7 inhibits the infiltration of macrophages and T cells, decreases the expression of CCL2, CXCL10, IL-1ß, IL17.	(141, 142)
Rheumatoid arthritis	IRF7 inhibits proinflammatory cytokine, promotes anti-inflamamtory cytokine IL-1ß.	(143)
Systemic lupus erythematosus (SLE)	IRF7 is a susceptibility locus, TRAP and Gfil prevent susceptibility to SLE by regulating nuclear transport of IRF7.	(106, 110, 144–154)
Systemic sclerosis( SSc)	The overexpression of IRF7 forms complexes with smad3, mediates the fibrosis.	(43, 155–158)
Autoimmune pancreatitis(AIP)	The IRF7-IFN-I-IL-33 axis mediates the development of AIP.	(159–162)
Autoimmune thyroid diseases (AITD)	IRF7 SNP is associated with increased susceptibility to AITD.	(163–165)
Diabetes	IRF7 interacts with Foxp3/CD8^+^T, affects the induction of TID, the STAT1-IRF7-MHC I complex axis accelerates the process of TID through IRF7-STAT2 cascade signals and promotes the proliferation of CD8^+^ T cells. IRF7 interacts with MCP-1 promotes the T2D development.	(166–172)

#### 3.2.1 IRF7 and MS/EAE

MS is a myelin-specific chronic inflammatory autoimmune disease. IRF7 is reduced in pDCs from patients with relapsing-remitting MS compared with healthy controls (148). In EAE, an animal model for MS, IRF7 deficiency resulted in more severe EAE, more infiltrating macrophages and T cells, and elevated levels of CCL2, CXCL10, IL-1β and IL17. The decreased expression of IRF7 represents a destructive function in MS/EAE (149).

### 3.2.2 IRF7 and RA

RA is characterized by persistent synovitis, systemic inflammation, and autoantibodies. In an arthritis model, IRF7 deficiency exacerbates the clinical severity, proinflammatory cytokines are increased, anti-inflammatory cytokine IFN-β is decreased, and IRF7 regulates the expression of IFN-β in murine macrophages but not in stromal fibroblast-like synoviocytes (150).

#### 3.2.3 IRF7 and SLE

SLE is characterized by the production of a variety of autoantibodies, complement activation and immune complex deposition, resulting in tissue and organ damage. Accumulating evidence implies that IRF7 is a susceptibility locus for SLE. IRF7 mRNA expression is significantly increased in SLE patients, and genetic polymorphisms near/in IRF7 have been substantiated to be related to the onset of SLE. Single nucleotide polymorphisms (SNPs) (rs191491376, rs1131665, rs1061501, rs4963128, rs702966, rs2246614) were found to be associated with SLE susceptibility, although some conflicting results were reported for the genetic heterogeneity between these populations (151–158). SLE patients treated with autologous stem cell transplantation show that high expression of IRF7 is correlated with recurrent lupus disease activity (159). For epigenetic modification, significant hypomethylation is observed in the IRF7 methylated site (160, 161). As mentioned above, TRAP and Gfi1 prevent susceptibility to SLE by regulating the nuclear transport of IRF7 (106, 110).

#### 3.2.4 IRF7 and SSc

SSc is a complex multisystem autoimmune disease that is characterized by widespread skin and internal organ fibrosis, immune system dysregulation, and vasculopathy. GWAS confirmed IRFs are genetic susceptibility loci in SSc (162). IRF5 SNP rs2004640 and rs2280714 are identified as a risk factor for SSc in whites and Asians., however rs4728142 is associated with lower IRF5 gene expression, longer survival and milder interstitial lung disease of SSc patients. The expression of IRF8 is decreased in SSc patients (163), IRF8 SNP rs11642873, rs2280381 and rs11117432 exhibit the strongest association with SSc risk (164–166). IRF4 SNP rs9328192 shows protective effect for SSc.

In addition to IRF5/IRF8/IRF4, IRF7 is regarded as a top predicted transcription factor of patients with SSc because increased expression is observed in peripheral blood cells (167, 168). The functional SNP rs1131665 leads to the activation of IRF7, and is associated with SSc risk for the presence of anticentromere autoantibodies (169). The methylation of IRF7 is associated with SSc, among the methylation status of 16 CpG sites at the promoter region of the IRF7 gene, CpG2 is significantly hypomethylated in SSc PBMCs and associated with increased disease risk, a significant difference in IRF7 mRNA expression between CpG8 methylated and unmethylated SSc patients, with four times higher in those who had a methylated CpG8 site than an unmethylated site (43). The mechanism may be that the overexpression of IRF7 in SSc fibrotic skin forms complexes with Smad3, the key component of TGF-β signaling for collagen production and fibrosis, and these complexes mediate fibrosis in dermal fibroblasts (170).

#### 3.2.5 IRF7 and AIP

IRF7 mediates AIP and human type 1 AIP (171). The authors previously reported that IFN-α and IL-33 produced by pDCs drive experimental AIP and human type 1 AIP (172–174). Now they disclosed that IRF7 activation and nuclear translocation is detected in AIP and human type 1 AIP, blockade of IRF7 signaling pathways decreased chronic fibroinflammatory responses via the suppression of pDC-mediated IFN-I and IL-33, the IRF7-IFN-I-IL-33 axis mediates the development of AIP.

#### 3.2.6 IRF7 and AITD

AITD is an organ-specific autoimmune disorder with immune attack on the thyroid and includes Graves’ disease (GD) and Hashimoto’s thyroiditis (HT), clinical hyperthyroidism and hypothyroidism (175, 176). IRF7 is a susceptibility gene for AITD, especially for GD and Graves’ ophthalmopathy. In a Chinese Han cohort, the IRF7 SNPs rs1131665 and rs1061502 were associated with increased susceptibility to GD, while rs1061501 was correlated with ophthalmopathy in GD patients (177). The function and mechanism of IRF7 in AITDs have not been clarified.

#### 3.2.7 IRF7 and diabetes

Type 1 diabetes (T1D) is characterized by autoimmune destruction of pancreatic β-islet cells, and fulminant type 1 diabetes (FT1D) is a subtype of idiopathic diabetes characterized by the absence of both insulitis and diabetes-related antibodies due to the destruction of pancreatic beta cells (178). IRF7 activated by TLR9 can bind to the Foxp3 core promoter and promote its transcriptional activity. The hypermethylated Foxp3 promoter blocks IRF7 binding to Foxp3, impairing the development and function of Tregs in FT1D patients, and deficient Tregs are prone to the development of FT1D (179). Using an IRF7^-/-^ bone marrow chimera model, Lang PA et al. found that the reduced expression of CD8^+^ T cells in pancreatic β-islet cells decreased the induction of T1D after LCMV infection (180).

In β cells, IFN-α promotes the nuclear translocation of STAT1 and IRF7, and the STAT1-IRF7-MHC I complex axis creates positive feedback through IRF7-STAT2 cascade amplifying signals and promotes the proliferation of CD8^+^ T cells, accelerating the process of T1D (181).

The IRF7-driven inflammatory network (IDIN) is enriched for viral response genes, and the human chromosome 13q32 locus controlling the IDIN was associated with the risk of T1D at the single nucleotide polymorphism rs9585056. Combined analyses of gene networks and DNA sequence variation implicated IRF7 network genes and their regulatory locus in the pathogenesis of T1D (182).

IRF7 deficiency prevents diet-induced obesity and insulin resistance (183). IRF7 transactivates MCP-1 by binding to its promoter in adipocytes, promoting the development of type 2 diabetes (184).

## 4 Conclusion and prospects

IFN signaling plays a causal role in host defense against infectious pathogenic organisms, the dysregulation is widely associated with autoimmune diseases, interferonopathy, infection, cancer and others, therefore, selective regulation of the IFN signaling may provide a therapeutic strategy. pDCs are the major producers of IFN- I, making them an appealing target for the treatment of autoimmune diseases. Litifilimab, a selected antibody against Blood dendritic cell antigen (BDCA) 2, a receptor is exclusively expressed on pDCs, shows efficacy in the treatment of SLE and cutaneous lupus erythematosus by decreasing the expression of IFN- I and ISGs (185, 186). IFN- I antagonists, such as anti-IFNα (sifalimumab, rontalizumab), anti- IFNαR(anifrolumab) appear effective in autoimmune disease, and can reduce ISG expression, the expression and nuclear translocation of IRF7 can be suppressed by the IFNα/β-Ab treatment (187–190). JAK inhibitors are approved for autoimmune, allergic diseases and most recently COVID-19 due to their potent efficacy in reducing IFN- I -driven inflammation. Tofacitinib, a pan-JAK inhibitor, used in the treatment of rheumatoid arthritis, ulcerative colitis et al, inhibits IFN- I production by suppressing transcription and nuclear translocation of IRF7, and affects DCs activities. Fedratinib, a JAK2 inhibitor, on one hand, can block new HIV-1 replication in acute HIV- I infection, on the other hand, can upregulate IRF7 transcription and phosphorylation, induce HIV-1 reactivation by an IFN-independent manner (191–193). As mentioned above, IRF7 is the main regulatory factor of IFN-I/III, small molecules can affect the transcription, translation, post translational regulation and nuclear transport of IRF7, thereby regulating the production of IFN. Therefore, IRF7 is expected to become an attractive therapeutic target for IFN-associated diseases.

IRF7 has multiple functions, as the vital step of the signaling pathway in IFN-I/III induction, the function and regulatory mechanism of IRF7 is important, which may help in understanding how to protect the host to reduce viral infection and maintain body balance. On the other hand, due to its tissue- and cell-specific and important role in autoimmune diseases, the relationship between IFN-I antagonists and IRF7, as well as the regulation of IRF7, is crucial for understanding the development of autoimmune diseases.

Although the role of IRF7 as a regulator of immune cell function has been extensively investigated, many questions remain unanswered and require explanation, such as the duality of IRF7’s role in conferring protection or exacerbation in different diseases, and the sophisticated signaling pathways require further elucidation. On the other hand, the role and mechanisms of IRF7 in IFN-III and autoimmunity remain to be explored. In addition, the expression, function and regulation of IRF7 in nonimmune cells remain unexplored, and more functions and mechanisms of IRF7 will be discovered with more in-depth studies in nonimmune cells. A deeper understanding of the precise functions and molecular mechanisms of IRF7 will be important for disease treatment.

## Author contributions

The manuscript was conceptualized by QG and WM. WM and QG wrote the manuscript. The figures were designed by GH and drawn by GH and ZW. LW summarized the tables. All authors contributed to the article and approved the submitted version.
